# Metagenome-assembled genome of withering syndrome causative agent, “*Candidatus* Xenohaliotis californiensis,“ from endangered white abalone (*Haliotis sorenseni*)

**DOI:** 10.1128/mra.00086-24

**Published:** 2024-04-30

**Authors:** Emily Kunselman, Sarah Allard, Colleen Burge, Blythe Marshman, Alyssa Frederick, Jack Gilbert

**Affiliations:** 1 Scripps Institution of Oceanography, Center for Marine Biotechnology and Biomedicine, La Jolla, California, USA; 2 Department of Pediatrics, University of California, La Jolla, California, USA; 3 California Department of Fish and Wildlife, Bodega Bay, California, USA; 4 Bodega Marine Laboratory, University of California Davis, Bodega Bay, California, USA; Montana State University, Bozeman, Montana, USA

**Keywords:** abalone, pathogen, genome

## Abstract

The genome of “*Candidatus* Xenohaliotis californiensis” was assembled from shotgun metagenomic sequencing of experimentally infected white abalone. Ninety-one percent genome completeness was achieved with low contamination. Sequencing this genome provides the opportunity to track pathogen evolution over time, conduct gene expression experiments, and study dynamics between this pathogen and its phage.

## ANNOUNCEMENT

White abalone (*Haliotis sorenseni*) from the Captive Breeding Program at UC Davis Bodega Marine Lab were experimentally infected with the intracellular bacterial pathogen, “*Candidatus* Xenohaliotis californiensis” (*Ca*Xc), using header tanks with infected abalone. *Ca*Xc is uncultured but can be transferred to other abalone through feces. *CaXc* presence and quantification were determined using *CaXc-*specific quantitative PCR (1) and 16S rRNA amplicon sequencing of the V4 region using primer pair 515F–806R (2). Post-esophagus, digestive gland tissue, and fecal matter were dissected, with some containing over 99% of *Candidatus* Xenohaliotis reads. One post-esophagus and one digestive gland sample from different abalone were chosen for shotgun metagenomic sequencing due to the high proportion of pathogen DNA. A fecal sample with 20% of *Candidatus* Xenohaliotis reads was also sequenced due to an expectation of lower host DNA contamination. The goal of shotgun metagenomic sequencing was to obtain a genome for the abalone pathogen, *Ca*Xc, which causes withering syndrome in many abalone species.

DNA was extracted by the UCSD Microbiome Core using the Applied Biosystems MagMax Ultra Nucleic Acid Isolation Kit (cat #A52358). Library preparation was conducted by the UCSD Microbiome Core using the KAPA Hyper Plus Kit (Roche Diagnostics, USA). Sequencing by synthesis was conducted by the UCSD IGM Genomics Center on the Illumina NovaSeq 6000 platform with paired-end 150 base pair cycles.

A protocol for assembling draft genomes from metagenomic sequencing reads was followed in KBase (3). Default parameters were used for all programs. Read quality was assessed with FastQC v0.12.1. A total of 18,524,162 sequences were obtained across all three samples, and sequence quality remained above 30 for all bases. Trimmomatic v0.36 was used to trim adapters and pair forward and reverse reads (4). Following this, 12,667,415 paired sequences were retained. metaSPAdes v3.15.3 was chosen for assembly based on best contig length, high N50, and low L50 (5). Contigs were binned using MaxBin2 v2.2.4, MetaBAT2 v1.7, and CONCOCT v1.1, and DAS tool v1.1.2 was used to optimize bins from all outputs combined (6–9). Three bins were generated, one of which was from the order Rickettsiales and was suspected to be the *Ca*Xc genome. Using QUAST (10, 11), this bin was found to have 35 contigs and N50 of 64,787.

To confirm the identity of the assembly as *CaXc*, the 16S rRNA gene sequence was extracted using ConTest16S (12) and was compared to partial *Ca*Xc sequences using NCBI’s BLASTn. The sequence shared 99% similarity across 99% of the query with “abalone withering syndrome agent” and 100% similarity across 88% of the query with “*Candidatus* Xenohaliotis californiensis.” This confirmed that the genome assembly is that of *Ca*Xc, the abalone pathogen in our experiment.

N’s in the *Ca*Xc assembly were removed before it was uploaded to MicroScope Microbial Genome Annotation and Analysis Platform v3.16.2 (13). The genome is 1,091,660 base-pairs long with 1,040 protein-coding sequences. CheckM (14) determined that the assembly had 91.1369% completeness and 1.18% contamination with 25 marker genes missing and three markers duplicated. *Ca*Xc is a gram-negative bacterium, and the genome has a guanine-cytosine content (GC) content of 30.99%.

## Data Availability

The *Ca*Xc genome is publicly available through ENA project accession PRJEB68339.
